# Sacroiliac Joint Pain in the Athlete

**DOI:** 10.31486/toj.21.0152

**Published:** 2022

**Authors:** Jacob Pfeiffer, Yuka Kobayashi, Andrew W. Gottschalk

**Affiliations:** ^1^ Department of Orthopedic Surgery, Medical College of Wisconsin, Milwaukee, WI; ^2^ Department of Orthopedic Surgery, Department of Family Medicine, Medical College of Wisconsin, Milwaukee, WI; ^3^ Department of Orthopedics, Ochsner Clinic Foundation, New Orleans, LA

## CASE PRESENTATION

A 52-year-old female amateur golfer presents to the sports medicine clinic with right-sided low back pain without inciting trauma. Her intermittent pain of several years' duration became more painful and persistent after a recent round of golf. She reports that she has a “bad back” and that formal physical therapy (PT) focusing on low back strengthening, 4 weeks of oral nonsteroidal anti-inflammatory drugs (NSAIDs), and activity modification have not been helpful in the past. Lumbar spine (anteroposterior, lateral) radiographs show degenerative changes at multiple levels without significant foraminal or spinal stenosis. Radiographs of the pelvis (anteroposterior, lateral, oblique) show mild degenerative changes of the bilateral sacroiliac joints (SIJs). On physical examination, she had positive FABER (flexion, abduction, and external rotation), Gaenslen, and compression tests.

## BACKGROUND

Low back pain is the top cause of disability globally, responsible for 60.1 million years lived with disability in 2015.^1^ That calculated disability number is a 54% increase from 1990 and accounts for more lost workdays than any other musculoskeletal condition in the United States.^1^ Low back pain, while a common complaint, can be difficult to accurately diagnose. One common etiology of low back pain is SIJ pain.^2^ An estimated 10% to 30% of patients with low back pain have SIJ-mediated pain.^2-5^ Given the social strain of medical expense and work productivity lost in this patient population, SIJ pain is an important pathology to understand and treat.

SIJ pain should be considered for athletes who present with complaints of low back pain because of the significant overlap in symptoms (Figure).^6^ Furthermore, 39% of patients diagnosed with SIJ pain have concomitant spinal pathology.^2^ To differentiate between spinal and SIJ pathology, the clinician is recommended to use multiple diagnostic maneuvers (Table).^7-9^ When 3 of the 6 maneuvers are positive, the sensitivity of diagnosis is 85% to 94%, and specificity is approximately 78%.^7-9^

**Figure.**
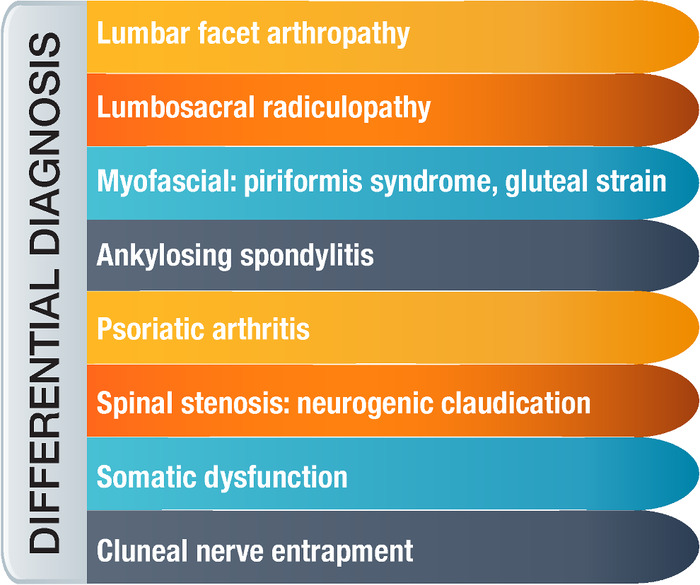
**Differential diagnoses for sacroiliac joint pain.** ^6^

**Table.** Diagnostic Maneuvers for Sacroiliac Joint Pain

Test	Description	Sensitivity, %	Specificity, %
FABER (Patrick)	With patient supine, bring ipsilateral leg into flexion, abduction, and external rotation while stabilizing the contralateral ASIS. Positive test is reproduction of posterior pain. Pain in the groin is suggestive of intra-articular hip pathology.^7,9^	35 to 69^9^	16 to 100^9^
Gaenslen	With patient supine, the ipsilateral leg and thigh hanging over the edge of table, and contralateral hip flexed, apply pressure downward on affected thigh and cranially to the flexed hip. Positive test is reproduction of pain in the sacroiliac joint region.^7-9^	21 to 68^8^	35 to >90^8^
Thigh thrust (posterior shear)	With patient supine, flex ipsilateral hip to 90°. Apply direct force through the femur posteriorly. Positive test is reproduction of or increased patient pain.^7-9^	42 to 80^8^	45 to 100^8^
Gapping (distraction)	With patient supine, place heels of both hands on each ASIS at the same time, directing force posteriorly and laterally. Positive test is reproduction of pain.^7-9^	15 to 90^8^	81^8^
Compression	With patient in lateral recumbent position with affected side up, flex hips to 45° and knees to 90°. Apply direct force downward on anterior superior iliac crest. Positive test is reproduction of posterior pain.^7-9^	7 to >60^8^	60 to 90^8^
Midline sacral thrust	With patient prone, apply pressure directly on the sacrum, with force anteriorly. Positive test is reproduction of pain.^8,9^	51 to >60^8^	40 to >60^8^

ASIS, anterior superior iliac spine; FABER, flexion, abduction, and external rotation.

Once SIJ pain is appropriately diagnosed, the goals of treatment are to manage symptoms and treat the underlying dysfunctions. The first steps for SIJ pain treatment in the athlete are activity modification, PT, manual manipulation, and short-term oral/topical medications.^7,8^ If patients fail to improve after conservative management, they can be considered for procedures including steroid injections, prolotherapy, radiofrequency denervation of the lateral branches, and sacroiliac joint stabilization and fusion surgeries.

## REVIEW OF EVIDENCE

### Physical Therapy

For athletes with core/hip weakness or lack of endurance, PT should be encouraged.^6^ In general, the goals of PT are core and pelvic stabilization, muscular imbalance corrections, and posture training.^2,6,7,10^ We interpret endurance in this context as the muscle strength needed to maintain pelvic neutral and core/pelvic stabilization during patient-dependent aggravating activities. In a study by Hungerford et al comparing 14 male subjects with SIJ pain to age-matched controls, researchers placed surface electrodes over 7 different hip and trunk muscles and measured the response and timing of muscle activation.^11^ The study showed significant delay of onset of the obliquus internus abdominis, multifidus, and gluteus maximus, with earlier biceps femoris electromyographic activity on the symptomatic side of those with SIJ pain.^11^ The authors proposed that the slowed firing of multiple hip and trunk muscles with earlier hamstring activation in the symptomatic group leads to suboptimal lumbopelvic stabilization that disrupts normal load transference through the pelvis and leads to SIJ pain.^11^ Up to 95% of patients with SIJ pain report good to excellent functional improvement with PT at 2-year follow-up.^12^

### Manual Manipulation

Manipulation is usually performed by osteopathic physicians, chiropractors, and physical therapists.^4^ A study of 69 patients showed that 95% of patients got excellent short-term results with manipulation; however, long-term benefits were not proven.^12^ Kamali and Shokri used 2 different manipulation protocols for patients with SIJ pain: one group received high-velocity low-amplitude (HVLA) treatments to the SIJ, and the other group received HVLA treatment to the SIJ and the lumbosacral spine.^4^ Both groups showed significant improvements on the visual analog scale (VAS) and Oswestry Disability Index (ODI)^13,14^ at 48 hours and 1 month postmanipulation.^4^ However, the study lacked a control group, as sham therapy is difficult to conduct.^4^ Appropriate patients must be chosen to undergo manipulation, as patients with significant muscle weakness, poor endurance, or both may need to focus on stabilization through strengthening rather than repetitive mobilization.^6^

### Medications

Evidence for the use of medications to treat SIJ pain is limited. Some evidence suggests a stepwise approach, starting with over-the-counter topicals (ie, lidocaine 5% 2 to 3 times daily, diclofenac 1% 2 to 4 times daily) and progressing to NSAIDs in the acute phase of SIJ pain (up to 6 weeks).^2,15^ NSAIDs (ie, ibuprofen 600 mg twice daily, diclofenac 50 mg twice daily) should not be used long term, specifically with senior athletes because of the significant side effects.^10^

### Injections

Injections are used both for diagnostic and therapeutic purposes in patients with SIJ pain.^16^ Corticosteroid injections are often considered the next step in treatment for athletes who do not improve with conservative measures or are unable to tolerate PT.^3,6,7,10,17^ Injections should always be done under image guidance (ultrasound or fluoroscopy) because intra-articular needle placement is rarely (0% to 22%) accomplished with blind injections.^5,18^ Jee et al compared fluoroscopy- to ultrasound-guided steroid injections with contrast in 120 subjects with noninflammatory sacroiliac arthritis and found fluoroscopic guidance to be more accurate than ultrasound guidance (98.2% vs 87.3%).^19^ However, after confirming placement of the needle, the researchers injected 1 mL of 0.5% lidocaine with dexamethasone 10 mg and found that the ultrasound and fluoroscopy groups had no statistical difference in improvement of pain on the VAS and ODI at 2- and 12-week follow-up.^19^

Kennedy et al found the effectiveness of SIJ injections to be highest in patients with ankylosing spondy-loarthropathies.^20^ For patients without the diagnosis of spondyloarthropathy, which is likely the case for most athletes with SIJ pain, the evidence for steroid injections is less clear.^20^ Studies investigating the efficacy of intra-articular injections in the SIJ often have differing outcome measures and medication protocols, so they are difficult to compare.^20^ For instance, most studies that used a single diagnostic anesthetic injection to diagnose patients with SIJ pain found patients had an average of 76 to 94 days of relief after their therapeutic SIJ injections; however, only 19% had lasting relief, and 32% had >14 days of relief.^20^

Comparatively, Liliang et al studied 150 patients who underwent dual SIJ block injections (2 intra-articular injections of 1 mL 0.5% bupivacaine or 0.2% lidocaine with 1 mL triamcinolone acetonide 40 mg) to diagnose and treat SIJ pain (39 patients were diagnosed).^21^ Patients’ pain and disability were assessed on the VAS and modified Oswestry Low Back Pain Disability Questionnaire.^21,22^ Statistically significant pain reduction was observed in mean VAS scores (*P*<0.0001) of the 39 patients diagnosed with SIJ pain. Of these patients, 66.7% had >50% pain reduction for more than 6 weeks, and 33% responded to the injection for only approximately 1 to 6 weeks with a mean of 4.4 ± 1.8 weeks.^21^ Of the patients who had >50% pain reduction for more than 6 weeks (n=26), their overall mean duration of pain reduction was 36.8 ± 9.9 weeks, with a range of 12 to 60 weeks.^21^

Complications related to SIJ injections are rare, but technical issues leading to unsuccessful injections (10% to 18%), temporary sciatic palsy (4.4% to 8%), increased pain (3%), and vasovagal reaction (2.5%) have been reported, as well as one case of pyogenic sacroiliitis and one reactivation of herpes simplex.^20^

More invasive procedures for chronic SIJ pain are outside the scope of this article, but these procedures include prolotherapy, radiofrequency denervation of the lateral branches (L5 dorsal ramus and lateral branches of the S1, S2, and S3 dorsal rami), and sacroiliac joint stabilization and fusion surgeries.^2,3,7^

## DISCUSSION

SIJ treatment should start with accurate diagnosis, followed by correction of biomechanical and muscle imbalances through PT.^12^ Image-guided diagnostic injections are a useful tool to quickly confirm the diagnosis, but the use of interventional treatments is reserved for patients who have refractory symptoms.^16^ The literature on the use of steroid injections to treat SIJ pain is difficult to summarize because (1) studies use single- vs dual-block diagnostic injections, (2) the types of medications and doses vary, (3) patient populations are not uniform, and (4) outcome measures differ across studies.^20^ Comprehensive double-blind studies are needed to understand the efficacy and appropriate use of image-guided injections to treat SIJ pain in the athlete.

## CASE RESOLUTION

After a complete history and physical examination, the athlete was diagnosed with SIJ pain. She started a formal, regimented course of PT once a week for 4 weeks to work on her core, pelvic, and hip strength. Three weeks into PT, she was unable to progress through her PT exercises because of pain, so a fluoroscopically guided SIJ injection was performed using 1 mL of 0.2% ropivacaine and 1 mL of triamcinolone acetonide 40 mg for diagnostic and therapeutic purposes. The patient noted she was able to progress better through PT 6 days after the injection because of improved pain control. At 6-week follow-up, the patient stated that her pain was 100% improved and that she is maintaining her home exercise strengthening program. She also noted she was able to golf on back-to-back days again.

## Notes

*This article meets the Accreditation Council for Graduate Medical Education and the American Board of Medical Specialties Maintenance of Certification competencies for Patient Care and Medical Knowledge.*
